# Diffusion potentials in saturated hardened cement paste upon chloride exposure

**DOI:** 10.1617/s11527-023-02184-y

**Published:** 2023-05-25

**Authors:** Elke Ziehensack, Sylvia Keßler, Ueli Angst, Harald Hilbig, Christoph Gehlen

**Affiliations:** 1 Centre for Building Materials, Technical University of Munich, Franz-Langinger-Straße 10, 81245 Munich, Germany; 2 Chair of Engineering Materials and Building Preservation, Helmut-Schmidt-University/University of the Federal Armed Forces Hamburg, Holstenhofweg 85, 22043 Hamburg, Germany; 3 Institute for Building Materials (IfB), Laura-Hezner-Weg 7, 8093 Zurich, Switzerland

**Keywords:** Diffusion potential, Permselective behavior, Concrete, Hardened cement paste, Chloride, pH

## Abstract

The diffusion potentials can cause significant errors in corrosion-related investigations of reinforced concrete structures (half-cell potential mapping, potentiometric sensors). Therefore, an improved understanding of the diffusion potentials in cement-based materials is needed. This study investigates the permselective behavior and its implication for the arising diffusion potentials. A diffusion cell is used to study the diffusion potentials in hardened cement pastes with imposed NaCl gradients. The cement pastes consist of ordinary Portland cement (OPC) and blast furnace cement (BFC) with water-cement ratios of 0.30–0.70. Laser ablation inductively coupled plasma mass spectrometry (LA-ICP-MS) is used to determine the concentration profiles of Cl, Na, K and Ca in the cement pastes with a high spatial resolution (100 µm). For the BFC pastes, considerable differences in the Cl^−^ and Na^+^ mobilities are found, indicating their permselective behavior. Despite the permselective behavior, the measured diffusion potentials are small (− 6 to + 3 mV) for all investigated cement pastes due to the high pH levels (13–14) in the pore solutions. However, when using the diffusion cell, the pH differences interfere with the measured diffusion potentials. The interfering pH differences need to be considered for an accurate measurement of the diffusion potentials in cement pastes.

## Introduction

### Effect of the diffusion potentials on the potential measurements in concrete

Durable reinforced concrete structures need to preserve their properties over their service lifetime. However, corrosion of reinforcing steel is one of the major deterioration mechanisms, which significantly degrades these structures.

Corrosion-related investigations in research and practice often rely on measuring the half-cell potential of steel embedded in concrete. Half-cell potential mapping is a well-established inspection method to localize corroding areas of embedded steel rebar [1–3]. This mapping is the basis for decisions on the maintenance and repair of reinforced concrete structures [1]. For monitoring systems, half-cell potential measurements are commonly used to detect corrosion onset in numerous research fields [4–10]. Furthermore, half-cell potential measurements enable the assessment of the effectiveness of corrosion protection and prevention methods, such as cathodic protection [11–13].

When studying the corrosion phenomena of embedded steel rebar, the transport of species in the concrete pore system is important. For ion transport investigations, the application of potentiometric sensors in concrete has gained increasing attention (e.g., chloride ion-selective electrodes and potentiometric pH sensors) [14–19]. These sensors monitor the chloride penetration and the pH decrease due to the progression of carbonation or leaching of concrete. They enable the nondestructive continuous measurements of the local chloride concentration and pH in the concrete pore solution.

All investigations require the correct evaluation of the potential readings to avoid misinterpretation of the measurement results [20, 21]. However, diffusion potentials in concrete interfere with these potential measurements and can act as a significant source of measurement error [13, 14, 20–24]. The diffusion potentials result from the diffusion and simultaneous charge separation of the ions in the concrete pore solution (compare [20, 21]). Whenever there are spatial differences in the composition of the concrete pore solution (e.g., chloride and pH differences), diffusion of Cl^−^ and OH^−^ and their accompanying cations (Na^+^, K^+^, Ca^2+^, Mg^2+^) occur. Simultaneous to ion diffusion, charge separation occurs due to the different mobilities of the diffusing anions and co-diffusing cations. The interfering diffusion potentials need to be quantified to enable the correction for the potential readings and thus accurate potential measurements. For investigations into chloride-induced steel corrosion, it is important to know the diffusion potentials in the chloride-contaminated concrete areas.

The diffusion potential can be calculated with the Henderson equation (Eq. 1).

1 $$E_{D} = \frac{RT}{F}\frac{{\sum u_{i} \frac{{\left| {z_{i} } \right|}}{{z_{i} }}\left( {c_{2,i} - c_{1,i} } \right)}}{{\sum u_{i} \left| {z_{i} } \right|\left( {c_{2,i} - c_{1,i} } \right)}}\ln \frac{{\sum u_{i} \left| {z_{i} } \right|c_{1,i} }}{{\sum u_{i} \left| {z_{i} } \right|c_{2,i} }}$$

2 $$\frac{{u_{i} }}{{D_{i} }} = \frac{{F\left| {z_{i} } \right|}}{RT}$$

where *c*_*1,i*_ and *c*_*2,i*_ denote the spatially different ion concentrations, *u*_*i*_ is the ion mobility, *D*_*i*_ is the diffusivity, *z*_*i*_ is the charge valence of species *i*, *T* is the temperature,* R* is the ideal gas constant and *F* is the Faraday constant (Eqs. 1–2 from [25]). The Henderson equation is suitable for estimating the diffusion potentials in solutions [20]. In concrete, the diffusion potentials have been estimated with Eq. 1 by adjusting the mobilities of the ions based on their diffusivities (Eq. 2) to account for the permselective behavior of concrete [23, 26]. Concrete shows permselective behavior if the diffusion of cations and anions through the pore system is affected to different extents (compare [21]). For the ion diffusivities, however, the data vary widely because there are many influencing factors originating from the water-cement ratio [27–29], the cement type [29, 30] and the moisture condition [31] of concrete. Therefore, a more accurate prediction of the diffusion potentials in concrete necessitates an enhanced understanding to adequately consider the permselective behavior.

### Measurement of the diffusion potentials in chloride-contaminated concrete

**Fig. 1:**
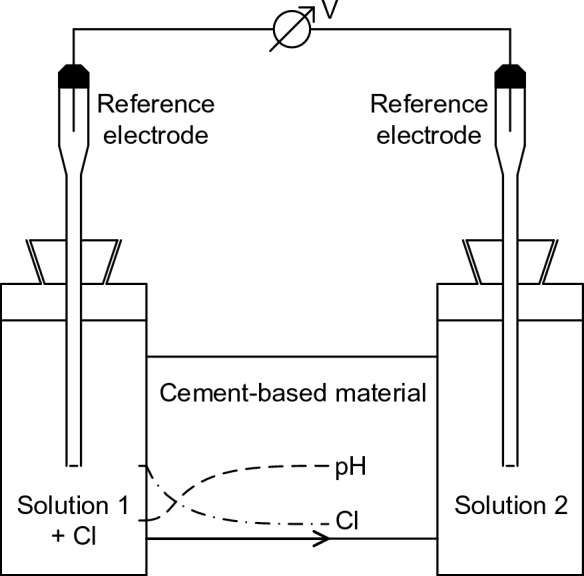
Diffusion cell setup for the study of the diffusion potentials in cement-based materials

When measuring the diffusion potentials in cement-based materials, the conventional diffusion cell setup has often been used [26, 32–35] (Fig. 1). In the diffusion cell setup, the material under study is placed between the two compartments filled with solution to impose the chloride gradient. The chloride solution usually consists of pure water with potassium chloride or sodium chloride. Owing to the generally high pH levels of 13–14 in the concrete pore solutions, there are pH differences of 6–7 units between the pore solution and the adjacent chloride solution in the compartment. This causes simultaneous leaching of hydroxyl ions from the pore solution and imposes a pH gradient in addition to the chloride gradient within the cement-based material under study. As several authors found a major contribution of pH differences to the resulting diffusion potential [20, 21, 23]; the overlap of the pH gradient with the chloride gradient can significantly affect the measured diffusion potential. Therefore, a more systematic study considering the contributions of the various parameters (ion concentration profiles, permselective property, etc.) to the overall diffusion potential is needed.

### Aim and scope of this study

The aim of this experimental study is to improve the current understanding of the diffusion potentials that arise in cement-based materials with imposed chloride gradients. This study examines the permselective behavior and its implication for the arising diffusion potentials. The diffusion cell setup is selected to study the diffusion potentials in saturated hardened cement pastes. Furthermore, solution experiments are conducted, and several setups are introduced for measuring the diffusion potentials in the solutions. The advantages and limitations of all measurement setups are discussed.

## Materials and methods

### Strategy and experimental program

**Fig. 2:**
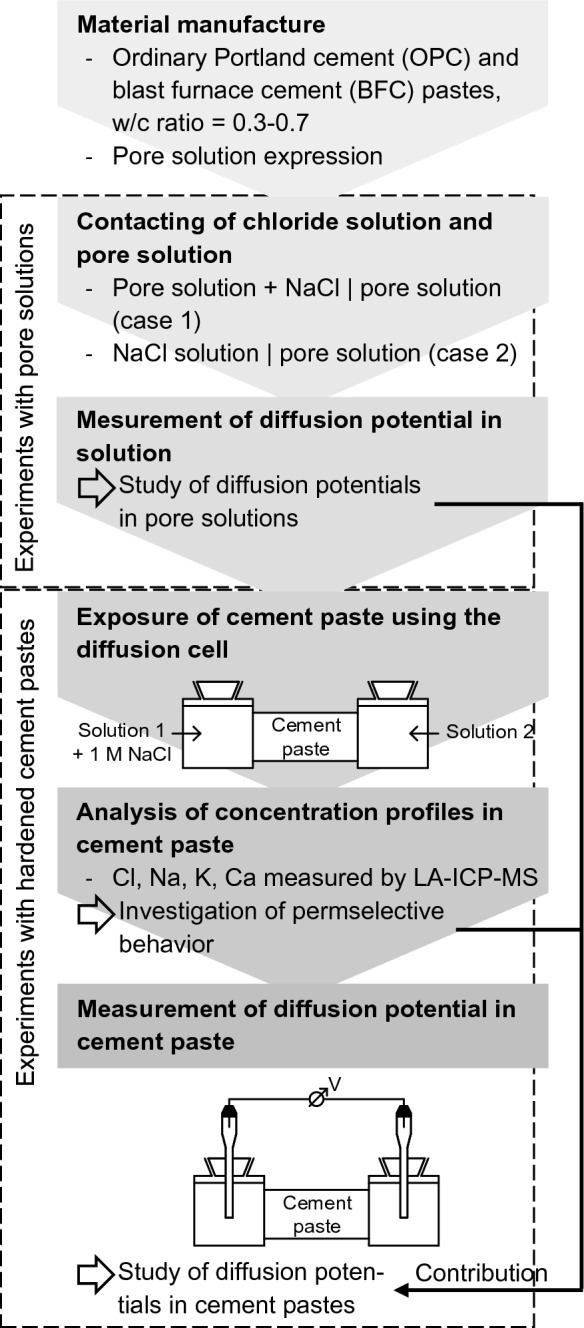
Experimental study on the diffusion potentials in saturated hardened cement pastes exposed to sodium chloride

Figure 2 shows the experimental setup used to measure the diffusion potentials in hardened cement pastes upon NaCl exposure. The diffusion cell setup was used to impose the chloride and sodium gradients in the cement pastes and to measure the arising diffusion potentials. Particular attention was given to minimize the pH differences at the junctions of the cement paste and the adjacent solutions 1 and 2, which interfered with the measured diffusion potentials. Synthetic pore solutions were filled in the diffusion cell compartments, and 1 M NaCl was added to solution 1. In this case, the imposed Cl^−^ and Na^+^ gradients overlapped with the high levels of pH and other species (K^+^, Ca^2+^, etc.) present in the pore solution (case 1). The effect of this on the arising diffusion potentials was studied in experiments with pore solutions to exclude the permselective behavior of the cement paste. The contribution of the permselective behavior to the arising diffusion potentials was determined by comparing how the diffusion potential in cement paste differed from the diffusion potential in pore solution. Based on a comparison with the conventional diffusion cell setup introduced in Sect. 1.2, additional solution experiments were conducted to consider the interfering pH differences (case 2).

The investigated cement pastes consisted of ordinary Portland cement (OPC) and blast furnace cement (BFC) with water-cement (w/c) ratios of 0.30–0.70. These cement pastes differed in their cement hydrate and pore solution chemistry and pore structure. While a wide range of pore solutions was investigated, the cement paste experiments focused on the cement pastes with w/c ratios of 0.30–0.50, which were expected to show permselective behavior. The temperature was approximately 20 °C during the experiments.

### Material manufacture and properties

**Fig. 3:**
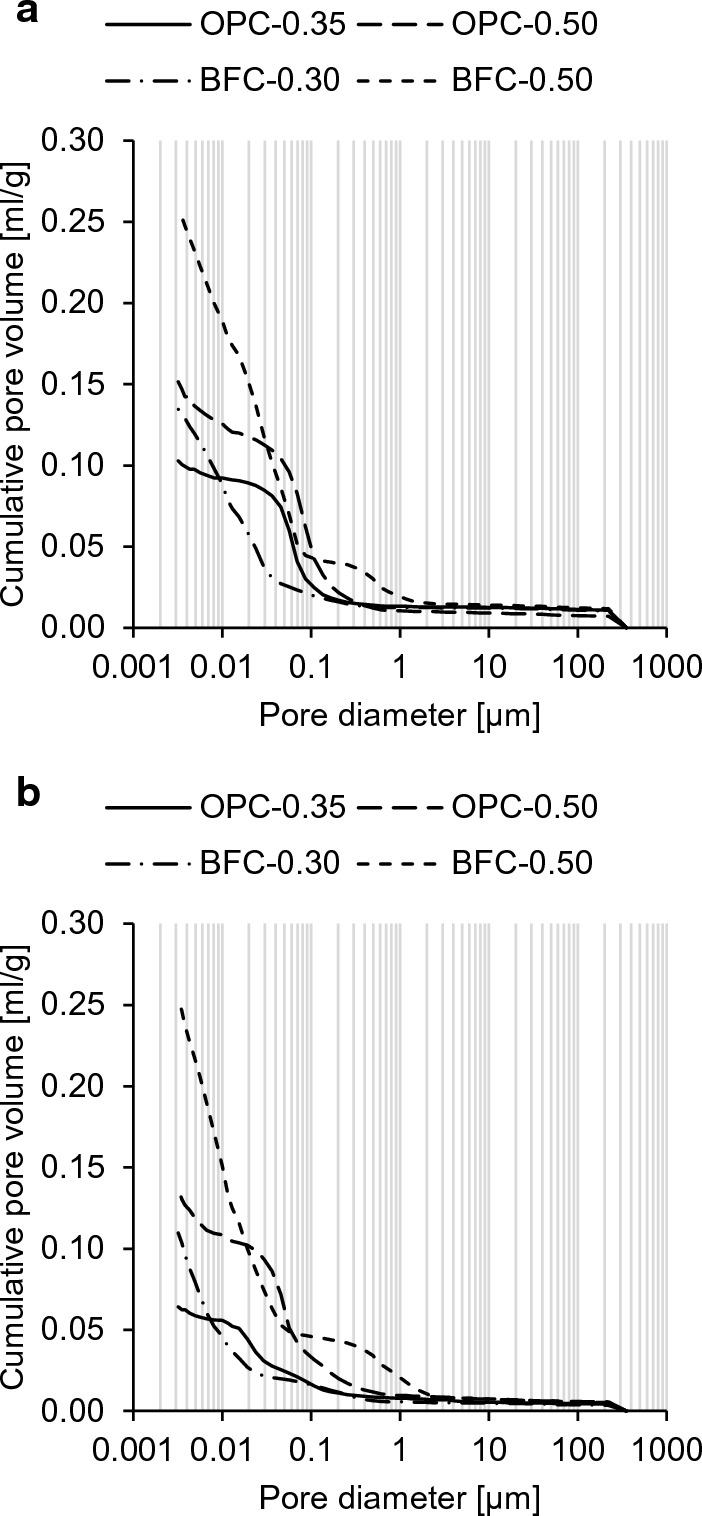
Cumulative pore volume of the studied OPC and BFC pastes at the ages of **a** 56 d and **b** 364 d as a function of the pore diameter

Table 1 shows the chemical compositions of the cements used and the mineralogical compositions of the hardened cement pastes (at 56 d) investigated in this study. The hydrofluoric acid-soluble species of the cements including the loss on ignition (LOI) were determined according to [36] and are provided in Table 1a. The BFC had a blast furnace slag proportion of 66–80 wt% according to [37]. All cement pastes rotated until they were hardened to prevent bleeding. The hardened cement pastes were cured in an airtight casting mold at room temperature. The mineralogical composition of the cement pastes was determined via X-ray diffraction (XRD)/Rietveld analysis (see Table 1b). Zinc oxide was used as an internal standard, and the data were normalized to 100 wt%. The pore structure of the cement pastes was determined via mercury intrusion porosimetry (MIP) at the ages of 56 d and 364 d to follow the shift toward smaller pore sizes with progressing hydration over the investigation period (Fig. 3) since this could have an effect on the permselective behavior.

**Table 1:** a Chemical compositions of the used cements; b Mineralogical compositions of the hardened cement pastes at 56 d

Cement	Content [wt-%]									Cl
Type	Oxide									
	SiO_2_	Al_2_O_3_	Fe_2_O_3_	CaO	MgO	SO_3_	K_2_O	Na_2_O	LOI	
*a*										
OPC	19.99	5.58	2.89	62.77	1.43	3.97	0.52	0.09	2.46	0.003
BFC	30.13	8.45	1.04	45.90	6.40	2.53	1.84	0.43	2.39	0.004
Portland cement [29]	18.94	5.65	3.29	63.2	3.13	3.43	0.78	0.34	1.13	
CEM I 52.5R (30%) + Slag 1 (70%) [52]	31.76	10.19	1.09	45.80	6.44	1.73	0.69	0.24	1.57	

	Cement paste				
	OPC-0.35	OPC-0.50	OPC-0.70	BFC-0.30	BFC-0.50
*b*					
Cement	OPC	OPC	OPC	BFC	BFC
w/c [−]	0.35	0.50	0.70	0.30	0.50
Phase content [wt-%]					
C3S	4.7	1.2	n.a.	2.5	1.8
C2S	8.6	5.0	n.a.	1.7	1.1
C3A	1.0	0.2	n.a.	–	–
C4AF	2.6	1.5	n.a.	–	–
Portlandite	13.0	16.7	n.a.	3.4	4.1
Ettringite	6.3	6.4	n.a.	5.0	5.0
Calcite	0.4	0.5	n.a.	2.6	2.3
Monocarbonate	6.0	6.8	n.a.	2.3	3.2
Amorphous phases	55.6	59.3	n.a.	81.8	80.0
Other phases	1.7	2.4	n.a.	0.6	2.6

For the investigation of the permselective behavior, the diffusion cell was used in a conventional way, as shown in Fig. 1. The cement paste samples had a diameter of 29 mm, and a length of 4 cm was selected for imposing the Cl^−^ and Na^+^ gradients over the investigation period of one year. The lateral sample surfaces were sealed with a polymer sleeve. After the curing period of 60 d, every sample was placed between the two diffusion cell compartments (compartment volume = 50 ml). Solution 1 was deionized water with 1 M NaCl, and solution 2 was deionized water without NaCl addition. Prior to NaCl exposure, deionized water without NaCl was added to both compartments over a period of 6 d for the conditioning of the sample.

Table 2a lists the compositions of the pore solutions expressed from the hardened cement pastes at the ages of 28 d and 91 d to monitor the changes in the pore solution compositions with ongoing hydration of the cement pastes. No significant changes were found, but a decrease in potassium and sulfide was observed in the BFC pastes. A maximum pressure of approximately 909 MPa was applied. In literature [38–40], the influence of the applied pressure on changes in the compositions of the expressed pore solutions has not been clarified. In addition to the expressed pore solutions, synthetic pore solutions were prepared using deionized water (see Table 2b). The pore solutions were stored in a CO_2_-free atmosphere to prevent carbonation.

**Table 2:** Compositions of a the pore solutions expressed from the hardened cement pastes at 28 d and 91 d, b the synthetic pore solutions

Cement paste	Age [d]	Ion concentration [M]						pH
		Na^+^	K^+^	Ca^2+^	Cl^−^	Sulfate	Sulfide	
*a*								
OPC-0.35	28	0.1231	0.5151	0.0006	< 0.001	0.0200	0.0098	13.76
	91	n.a.	n.a.	n.a.	n.a.	n.a.	n.a.	n.a.
OPC-0.50	28	0.0753	0.2943	0.0008	< 0.001	0.0032	0.0029	13.56
	91	0.0748	0.2963	0.0024	< 0.001	0.0097	0.0000	13.57
OPC-0.70	28	0.0531	0.1899	0.0018	< 0.001	0.0007	0.0009	13.38
	91	0.0527	0.1923	0.0030	< 0.001	0.0040	0.0000	13.40
BFC-0.30	28	0.1128	0.3585	0.0004	0.032	0.0190	0.1163	13.60
	91	0.1115	0.3312	0.0010	0.025	0.0182	0.0072	13.61
BFC-0.50	28	0.0740	0.1951	0.0004	0.006	0.0039	0.1374	13.41
	91	0.0738	0.1889	0.0016	0.008	0.0061	0.0055	13.40

Cement paste	Content^a^ [g/l]				pH^b^
	KOH	NaOH	Na_2_SO_4_	KCl	
*b*					
OPC-0.35	19.701	3.559	1.189	0.040	13.43
OPC-0.50	11.041	2.441	0.331	0.032	13.23
BFC-0.30	6.183	0.325	2.154	0.750	12.97
BFC-0.50	4.312	0.874	0.589	0.592	12.90

^a^Synthetic pore solutions were saturated with Ca(OH)_2_

^b^Measurement with pH meter

### Diffusion potential measurement

**Fig. 4:**
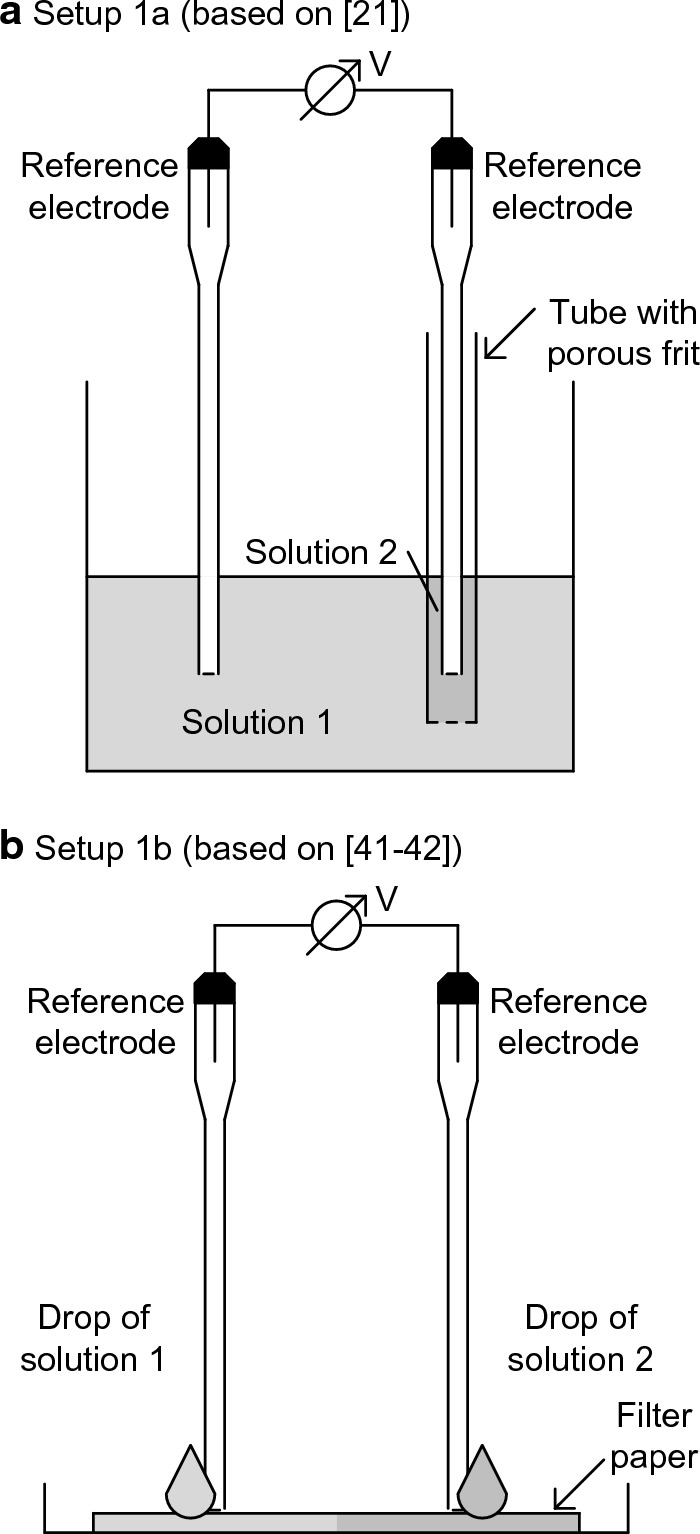
Diffusion potential measurement setups in the pore solution experiments; **a** setup 1a with porous rit in the tube and **b** setup 1b with filter paper

Figure 4 shows the setups used for the diffusion potential measurement in the pore solution experiments. In both setups, solution 1 and solution 2 were of different compositions and came into direct contact using a porous membrane. The diffusion potential that occurred at the junction of solution 1 and solution 2 was measured. In setup 1a (used in, e.g., [21]), a porous frit separated solution 1 and solution 2. In setup 1b, which was used in [41, 42], a drop of each solution was applied on a piece of cellulosic filter paper at the left/right reference electrode. Then, both solutions moved toward the center, where a concentration gradient across the junction region was established, and thus, a diffusion potential occurred. The performance of setups 1a and 1b was examined using NaCl and NaOH solutions with various concentrations ranging from 0.00001 to 1 M in deionized water.

When measuring the diffusion potentials in the cement pastes, the diffusion cell setup in Fig. 1 was used with the following modifications. Synthetic pore solutions were used in the diffusion cell compartments with the addition of 1 M NaCl to solution 1, as particular attention was given to minimize the interfering pH differences. The cement paste samples had a diameter of 29 mm, and a length of 3 cm was sufficient for imposing the Cl^−^ and Na^+^ gradients since pH values of 13–14 in the chloride solution were assumed to considerably reduce the Cl uptake (compare [43, 44]). For the OPC pastes, a curing period of 35 d was selected, whereas the curing period was 59 d for the BFC pastes considering their slower hydration. The influence of the different curing periods on the measured diffusion potentials were considered to be negligible because there were no significant changes in the pore solution compositions between 28 and 91 d. As can be seen in [45], the pore solution composition determined the measured diffusion potentials in the cement pastes at the beginning of the NaCl exposure, while the contribution of the permselective behavior was negligible. Prior to NaCl exposure, both compartments were filled with synthetic pore solutions without NaCl addition over a period of 7 d to condition the sample. The solutions in both compartments were renewed before each diffusion potential measurement.

The diffusion potentials were measured by means of a high impedance voltmeter and a pair of Ag/AgCl/KCl_sat_ reference electrodes (RE). Stable potential readings were recorded, i.e., ≤ 0.2 mV/min. The use of these reference electrodes included additional diffusion potentials at the junction of the inner electrolyte of the reference electrode and solution 1 or 2. The potential readings were corrected by the residual electrode potential difference and the diffusion potentials due to the coupling of the reference electrodes, which were estimated with Eq. 1.

### Concentration analysis of the species and their profiles

Several analyses were used to determine the acid-soluble species in the cement pastes and the compositions of the solutions (expressed pore solutions, the solutions in diffusion cell compartments).

For the analysis of the solution compositions, 0.1–0.5 ml sample solution was diluted with deionized water (1:50). Na, K, Ca and S were analyzed via inductively coupled plasma optical emission spectrometry (ICP-OES) according to [46]. The used ICP-OES equipment was a PerkinElmer Avio 500. Cl was determined via potentiometric titration with 0.1 M AgNO_3_ for concentrations > 500 mg/l; otherwise, photometric determination was performed using a Cuvette Test System LCK 311 (Hach). Sulfate was analyzed via ion chromatography (IC). Sulfide was calculated by subtracting the proportion of sulfate from the total sulfur concentration. For the pH analysis, undiluted sample solutions were used. The pH was measured with a universal indicator for values ≤ 11. For values > 11, the pH of 0.5 ml solution was determined by titration with 0.1 M HCl using a neutral red indicator.

The cement pastes were pulverized in a ball mill (5 min) and dried at 105 °C (2 h). The acid-soluble species in the cement pastes were extracted in 3 M HNO_3_ solution (0.5 g cement paste in 25 ml HNO_3_ solution) according to [47]. For the analysis, the extraction solution was diluted with deionized water (1:10). Na, K, Ca and S were analyzed via ICP-OES. Cl was determined as described for the above solutions. Sulfate was calculated on the basis of the total sulfur concentration.

The concentration profiles of the species in the cement pastes were investigated by laser ablation inductively coupled plasma mass spectrometry (LA-ICP-MS). LA-ICP-MS was shown to be suitable for analysis of cement-based materials (see, e.g., [48–50]). This method enabled the analysis of the spatial distributions of the species with a high resolution in the micrometer range. The depth resolution was estimated to be 10–20 µm and usually depends on the measurement settings and the material under study. The LA-ICP-MS equipment used was an ESI NWR213 laser ablation system (Nd:YAG, wavelength = 213 mm, pulse width = 4–6 ns, laser fluence = 2.0 J/cm^2^, fully Q-switched mode) coupled to a PerkinElmer NexION 300D ICP-MS. Before measurement, the samples from the diffusion cell (see Sect. 2.2) were divided along their longitudinal axis via sawing without the use of water to avoid washing out the species from the cement paste. Then, the samples were dried with the help of silica gel granules over a period of 14 d because the high moisture contents could have interfered with the LA-ICP-MS measurements (compare [50]). After preablation, the laser was shot on the sawed sample surface (spot diameter = 100 µm, repetition rate = 20 Hz) while moving with a scan speed of 30 µm/s in the longitudinal direction to obtain the species concentration profiles. The carrier gas consisted of 0.7 l/min He and 0.92 l/min Ar. The measured isotopes were ^13^C, ^23^Na, ^26^ Mg, ^27^Al, ^29^Si, ^34^S, ^35^Cl, ^39^ K, ^43^Ca and ^54^Fe.

**Fig. 5:**
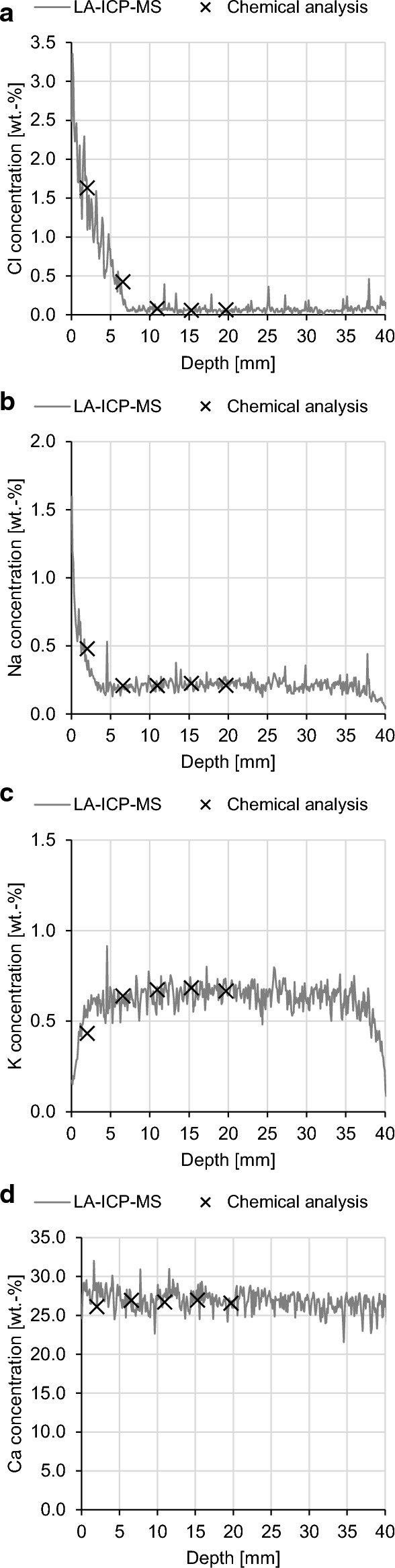
Calibration of **a** Cl, **b** Na, **c** K and **d** Ca profiles (resolution = 100 µm) measured by LA-ICP-MS using the concentration profiles of the acid-soluble species

The LA-ICP-MS signal intensities were converted to net signal intensities after subtraction of the corresponding background signal. To convert the signal intensities to species concentrations, the samples were further divided into disks with a thickness of 3–5 mm via sawing without the use of water. The concentrations of the nitric acid-soluble species were used to calibrate the LA-ICP-MS signals. Figure 5 shows the calibration of the Cl, Na, K and Ca profiles in a BFC paste sample (w/c = 0.5) after 360 d of NaCl exposure.

### Determining the bound/free Cl and the diffusion coefficients of Cl and Na

The concentration profiles of the dissolved (free) Cl^−^ in the cement pastes were determined with Eqs. 3–5 (from [51]). Bound Cl was calculated with a Freundlich binding isotherm (Eq. 5).

3 $$C_{t} = C_{f} + C_{b}$$

4 $$C_{f} = V_{p} \cdot c_{f}$$

5 $$C_{b} = \alpha \cdot c_{f}^{\beta }$$

where *C*_*t*_, *C*_*f*_, *C*_*b*_ [*g*_Cl_/*g*_paste_] are the total, free and bound Cl concentrations in cement paste, respectively; *V*_*p*_ [dm^3^/g] is the pore volume; *c*_*f*_ [*g*_Cl_/*l*_solution_] in Eq. 4, *c*_*f*_ [mol/l] in Eq. 5 is the free Cl concentration in pore solution. Data for *α* and *β* were used from the literature (*α* = 9.86 and *β* = 0.24 [29] for the OPC pastes; *α* = 21.87 and *β* = 0.55 [52] for the BFC pastes), in which the cement compositions were found to be similar to those in our study (see Table 2a).

The concentration profiles enabled the determination of the diffusion coefficients by least squares curve fitting using nonlinear regression. The regression based on Fick’s second law of diffusion using Eq. 6 (from [53, 54]).

6 $$C\left( {x,t} \right) = C_{0} + \left( {C_{s} - C_{0} } \right)\left( {1 - erf\left( {\frac{x}{{2\sqrt {D \cdot t} }}} \right)} \right)$$

where *C(x,t)* [*g*/*g*_paste_] is the total/free concentration of Cl and Na at the sample depth *x* [m] and exposure time *t* [s]; *C*_*0*_ [*g*/*g*_paste_] is the initial concentration; *C*_*s*_ [*g*/*g*_paste_] is the concentration at the exposed sample surface; *D* [m^2^/s] is the diffusion coefficient. The least squares curve fitting was carried out by using Eq. 7.

7 $$\mathop \sum \limits_{i = 1}^{n} \left( {C_{i} - \hat{C}_{i} } \right)^{2} = \min$$

where *C*_*i*_ is the measured concentration value, and *Ĉ*_*i*_ is the corresponding value calculated with Eq. 6. Both the apparent (*D*_app_) and effective (*D*_eff_) diffusion coefficients were determined. *D*_app_ and *D*_eff_ based on the profiles of total concentrations of Cl and Na measured by LA-ICP-MS with a resolution of 1 mm and the profiles of free species concentrations, respectively (*R* > 0.9).

## Results and discussion

### Experiments with the pore solutions

#### Performance of the diffusion potential measurement setups

Several setups are available for measuring the diffusion potentials in solutions. Two of them are used in our study, and further setups can be found in the literature (e.g., [20, 54–58]). In every setup, there is the junction of two solutions. The generation of this junction is challenging because the two solutions have to come into contact without or with limited convective mixing (compare [57]). One option is the use of a porous membrane [20–22, 41, 42]. However, this requires a membrane that does not have permselective behavior, which can affect the arising diffusion potential. Therefore, the performance of each measurement setup needs to be examined in this regard before use.

**Fig. 6:**
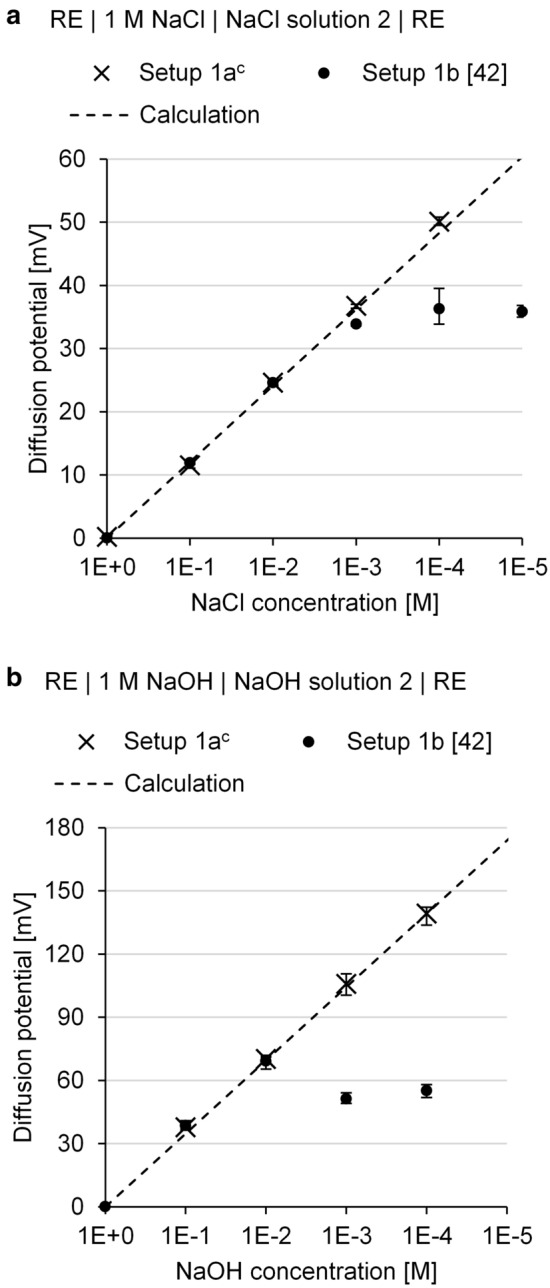
Measured and calculated diffusion potentials as a function of the **a** NaCl concentration and **b** NaOH concentration in solution 2; mean, minimum and maximum value of three independent measurements. ^c^No stable potential reading at a concentration of 0.00001 M

Figure 6 plots the diffusion potentials for NaCl and NaOH solutions as a function of the concentration for solution 2. The concentration in solution 1 is kept constant at 1 M. This figure shows the measured diffusion potentials using setups 1a and 1b and the diffusion potentials calculated with Eq. 1. It compiles both the measurement results from our study and from the literature [42]. The diffusion potential linearly increases with magnitudes of 12 mV and 35 mV per decade of NaCl concentration and NaOH concentration, respectively. The deviation of three independent measurements is usually low. This indicates good repeatability of the diffusion potential measurements. When using different setups, the measured diffusion potentials are found to be comparable for NaCl concentrations of up to 0.001 M and for NaOH concentrations of up to 0.01 M.

Setup 1a enables measurements at concentrations of up to 0.0001 M, and the results deviate for the setup with filter paper (setup 1b). An extraction test of the filter paper shows that the soluble species in the filter paper contaminate the studied solutions. In this test, the filter paper used was stored in deionized water over a period of one day. The ionic conductivity of the water measured 264 µS/cm at the end of the storage period compared to the uncontaminated deionized water with a conductivity of 0.7 µS/cm at the same temperature of 22.1 °C. The contamination particularly affects the ionic strength of dilute solutions and depresses the diffusion potentials measured with setup 1b at very low NaCl and NaOH concentrations [42]. However, its effect is negligible for concentrations larger than 0.001 M NaCl and 0.01 M NaOH. Therefore, setup 1b can reliably be used for the diffusion potential measurement within the above concentration ranges.

There is good agreement between the diffusion potentials measured with the various setups and with the calculated diffusion potentials. This demonstrates that the used frit and filter paper do not show permselective behavior for the studied species and concentration ranges. Thus, setups 1a and 1b enable diffusion potential measurement without interference from permselective action and are suitable for pore solution experiments. For these experiments, setup 1a is selected since the use of setup 1b has some limitations.

#### Diffusion potentials in the pore solution

The study of the diffusion potentials in the pore solutions covers case 1 and case 2 (see Fig. 2). In both cases, solution 1 is the chloride solution, and solution 2 is the pore solution. To reproduce case 1, solutions 1 and 2 are synthetic pore solutions with 1 M NaCl added to solution 1. This only imposes a difference in the Cl^−^ and Na^+^ concentrations and excludes concentration differences for all other species (K^+^, Ca^2+^, etc.) present in the pore solution, including the pH. To reproduce case 2, solution 1 is deionized water with various NaCl concentrations ranging from 0.001 to 1 M, and solution 2 is the expressed pore solution. In this case, pH differences of 6–7 units overlap with the Cl^−^ and Na^+^ differences.

**Fig. 7:**
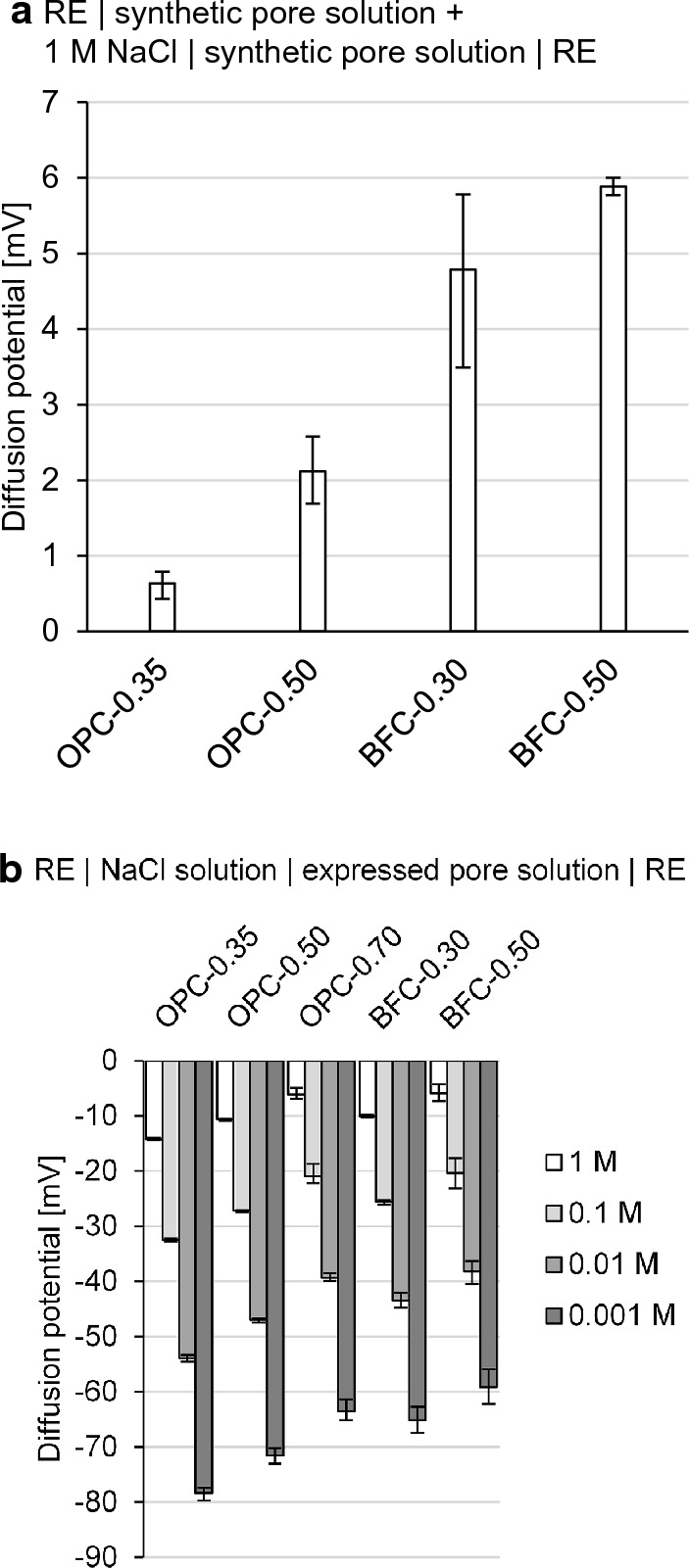
Measured diffusion potentials at the junction of solution 1 and solution 2 using setup 1a for **a** case 1 and **b** case 2; mean, minimum and maximum value of three independent measurements

Figure 7a and b show the measured diffusion potentials that arise at the junction of solution 1 and solution 2 using setup 1a for case 1 and case 2, respectively. In Fig. 7a, the diffusion potentials are not higher than 6 mV despite the large Cl^−^ and Na^+^ differences of 1 M. The diffusion potentials decrease slightly with decreasing w/c ratio for both the OPC and BFC pore solutions. This correlates with the increase in the pH level in the pore solutions. For comparison, when the pH level is approximately 7 in water and there is a NaCl concentration difference of 1 M, the diffusion potentials are much higher than 50 mV (see Fig. 6a). The high pH levels of 13–14 in the pore solutions are associated with a high ionic strength, which depresses the arising diffusion potentials, as shown in [20, 23]. The higher pH levels in the OPC pore solutions depresses the diffusion potentials much more than those in the BFC pore solutions.

In Fig. 7b, diffusion potentials of up to − 78 to − 64 mV occur for the OPC pore solutions, while the resulting values are − 65 to − 59 mV for the BFC pore solutions. With increasing w/c ratio, the diffusion potentials change to more positive values (smaller absolute diffusion potentials) for both the OPC and BFC pore solutions. This correlates with the decreasing pH values of the pore solutions and thus smaller pH differences at the junction. With increasing NaCl concentration, the diffusion potentials decrease in size. This is attributed to the increasing ionic strength in the NaCl solution, which depresses the diffusion potentials, as shown by Angst et al. [21]. For a NaCl concentration (difference) of 1 M, the diffusion potentials measured are − 15 to − 5 mV. Compared to case 1, negative diffusion potential values result owing to the pH differences.

In both cases, the diffusion potentials are considerably affected by the different pore solution compositions (particularly the OH^−^ concentrations) of the various cement pastes (differences of up to 19 mV). This shows the high pH sensitivity of the arising diffusion potential and supports the results in [26].

### Experiments with the cement pastes using the diffusion cell

#### Permselective behavior of the cement paste

**Fig. 8:**
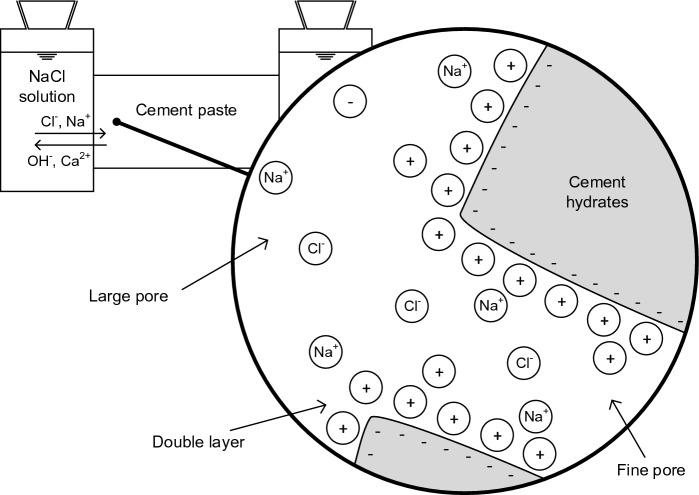
Illustration of the chloride and sodium ion diffusion in the pore system of the cement paste with permselective behavior; cations (+) and anions (−) initially being present in the pore solution and penetrating sodium ions (Na^+^) and chloride ions (Cl^−^)

When studying the diffusion potentials in cement pastes, it is important to examine whether the cement paste affects the diffusion of Cl^−^ and Na^+^ to different extents (permselective behavior). The investigation of the permselective behavior requires a holistic view of the diffusion of Cl^−^ and Na^+^ through the cement paste, including the pore structure, ion binding and pore solution composition. Figure 8 shows the diffusion cell, in which the cement paste is exposed to sodium chloride. This figure illustrates the diffusion of Cl^−^ and Na^+^ in the pore system of a permselective cement paste. The penetrating Cl^−^ and Na^+^ interact with the ions present in the pore solution and with the solid hydrated phases on the pore surface.

Owing to the electronegative character of the C–(A–)S–H phase [59], an electrical double layer forms on the pore surface to balance the charge [60]. The negatively charged pore surface attracts cations (mainly Ca^2+^), which preferentially accumulate near the surface, whereas anions remain farther away [60]. This can reduce the mobility of particularly the cations in the double layer and thus slows down their diffusion process [24]. The thickness of the double layer is estimated to be 1–2 nm because of the high ionic strength of the concrete pore solutions [24, 60]. While the double layers of the opposite pore walls can overlap in fine pores, the charged surface almost does not influence the pore solution in large water-filled capillary pores [24]. Eventually, this does not significantly affect the diffusion of the penetrating Cl^−^ and Na^+^ in the bulk solution of large pores. However, when the fine pores interconnect the large capillary pores, they can have a more pronounced effect on slowing down the penetration of Na^+^ [24]. Therefore, Angst et al. [24] expect the permselective behavior at low water-cement ratios, high degrees of hydration and low moisture contents with only the small pores being filled with pore solution.

**Fig. 9:**
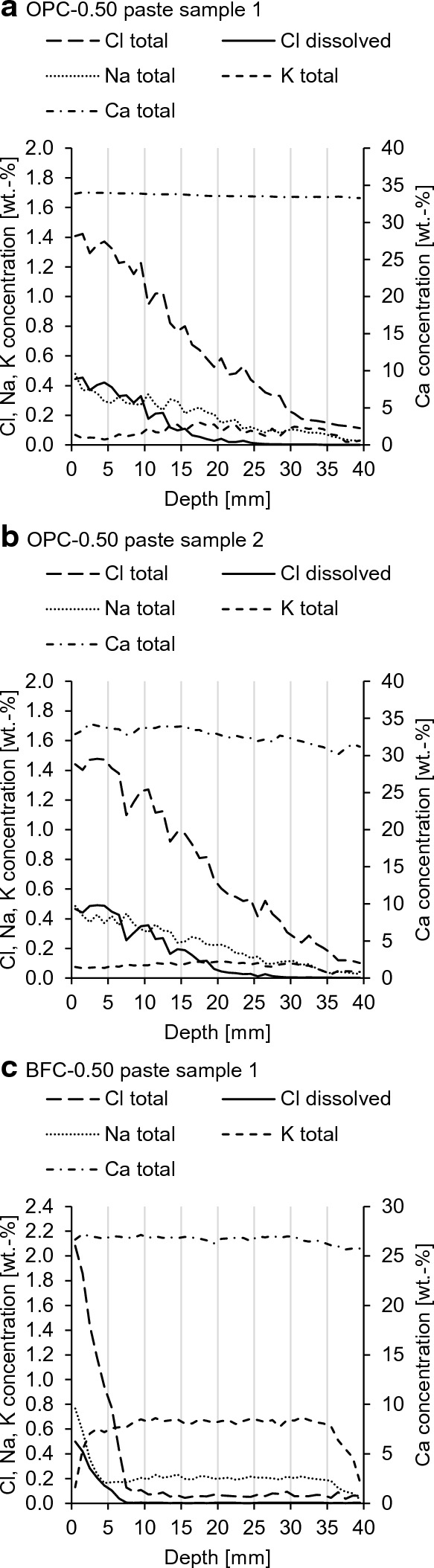

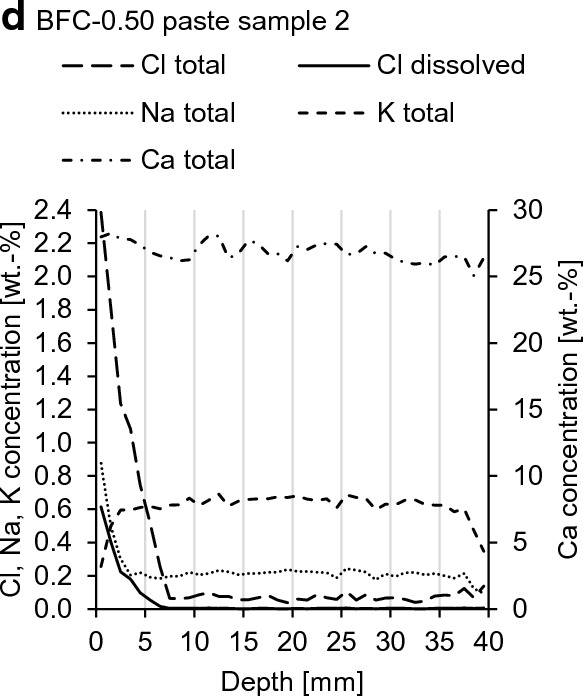
Profiles of total concentrations of Cl, Na, K and Ca and dissolved (free) Cl^−^ in the **a**, **b** OPC-0.50 and **c**, **d** BFC-0.50 pastes after 360 d NaCl exposure (resolution = 1 mm)

Figure 9 plots the concentration profiles of Cl, Na, K and Ca in the cement pastes after 360 d of NaCl exposure. It shows the profiles of the total species concentrations measured via LA-ICP-MS and the dissolved (free) Cl^−^ calculated with Eqs. 3–5. The profiles show the penetration of Cl and Na and the simultaneous leaching of K and Ca. Cl penetrates the OPC-0.5 paste almost completely, while the Cl penetration of the BFC-0.5 paste is approximately 7.5 mm. Compared to Na, Cl penetrates deeper into the cement paste. The faster penetration of Cl than Na has also been found in the literature [60–64].

For comparison, Table 3 shows the corresponding diffusion coefficients of Cl and Na in the cement pastes obtained with Eq. 6 and the intrinsic diffusion coefficients for dilute bulk solutions. For BFC-0.50, *D*_app,Na_ is smaller than *D*_app,Cl_ by one order of magnitude, while this difference is not as pronounced for OPC-0.5. The *D*_app,Cl_/*D*_app,Na_ ratio for OPC-0.50 is similar to the *D*_Cl_/*D*_Na_ ratio in solution, whereas the *D*_app,Cl_/*D*_app,Na_ ratio for BFC-0.50 is considerably higher. With regard to the arising diffusion potentials, the concentrations of the dissolved Cl^−^ and Na^+^ are relevant rather than their total concentrations. As negligible Na^+^ binding is expected for the OPC-0.5 paste (compare [65]), the profiles of total and dissolved Na^+^ are assumed to be equal, i.e., *D*_app,Na_ is equal to *D*_eff,Na_. For the BFC-0.5 paste, however, Na^+^ binding is presumed to occur due to the higher amount of aluminate-bearing phases (compare [66]), i.e., *D*_eff,Na_ is even smaller than *D*_app,Na_.

**Table 3:** Diffusion coefficients of Cl and Na in the cement pastes and intrinsic diffusion coefficients for dilute bulk solutions

Material	Species	Diffusion coefficient [*10^–12^ m^2^/s]			Ratio [−]	
		*D*	*D*_app_	*D*_eff_	*D*_Cl_/*D*_Na_	*D*_app,Cl_/*D*_app,Na_
Solution [68]	Cl	2.03 * 10^3^			1.5	
	Na	1.33 * 10^3^				
OPC-0.50 paste	Cl		8.07 ± 0.69	2.94 ± 0.40		1.3–1.5
	Na		5.80 ± 0.12			
BFC-0.50 paste	Cl		0.30 ± 0.06	0.18 ± 0.05		6.0–6.9
	Na		0.05 ± 0.01			

The significantly reduced mobility of Na^+^ compared to Cl^−^ in the BFC-0.50 paste can be explained as follows. There is a high amount of pore volume accessible through fine pores with a few nanometers (< 10 nm) in the BFC-0.50 paste (see Fig. 3). Although MIP is restricted to pore sizes of 0.003–350 µm, it is assumed that there are pores smaller than 3 nm. This indicates that the double layer slows down Na^+^ and thus reduces the mobility of Na^+^ much more than that of Cl^−^. For BFC-0.30, the effect on slowing down Na^+^ is expected to be even more pronounced due to the higher amount of fine pores.

**Fig. 10:**
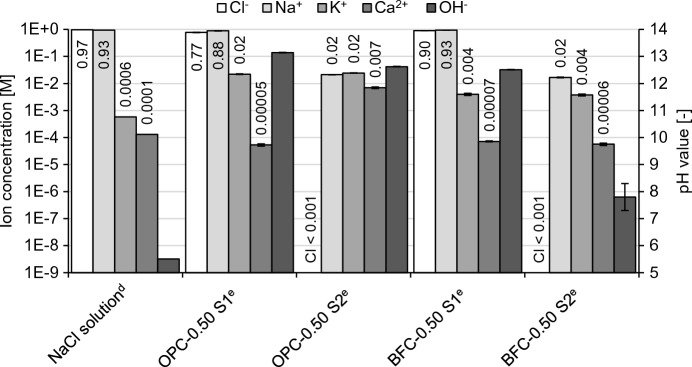
Compositions of solution 1 (S1) and solution 2 (S2) in the diffusion cell compartments after 360 d NaCl exposure and the NaCl solution before NaCl exposure. ^d^Single value, ^e^Mean, minimum and maximum value of two independent measurements

Figure 10 shows the compositions of the solutions in the diffusion cell compartments after 360 d of NaCl exposure and the NaCl solution before NaCl exposure. The actual concentrations of Na^+^ and Cl^−^ in the NaCl solution are slightly lower than 1 M. The Cl^−^ concentrations decrease by 21% in OPC-0.5 S1 and by 7% in BFC-0.5 S1, whereas the Na^+^ concentrations decrease to a much lower extent. The pH values increase to 13.1 and 12.5 in OPC-0.5 S1 and BFC-0.5 S1, respectively, and K^+^ and Ca^2+^ are found. In both S1 solutions, the concentrations of OH^−^ are higher than those of K^+^ by one order of magnitude. This shows that there is the exchange of Cl^−^ with OH^−^ rather than the movement of the cations. This supports the results in Johannesson et al. [61] that the leaching of OH^−^ from the pore solution was faster than that of Ca^2+^, which was located closer to the negatively charged pore surfaces and therefore less mobile. They concluded that this drastically hindered Na^+^ from penetrating the concrete. This is in accordance with the results from this study; specifically, a higher chloride uptake and a faster chloride penetration of both cement pastes compared to sodium are found.

These investigations show that there are considerable differences in the Cl^−^ and Na^+^ mobilities for the investigated BFC pastes and indicate the permselective behavior of these pastes, whereas this difference is not as pronounced for OPC-0.5. Additional investigations are required for OPC-0.35 to confirm the permselective behavior.

#### Diffusion potentials in the cement paste

**Fig. 11:**
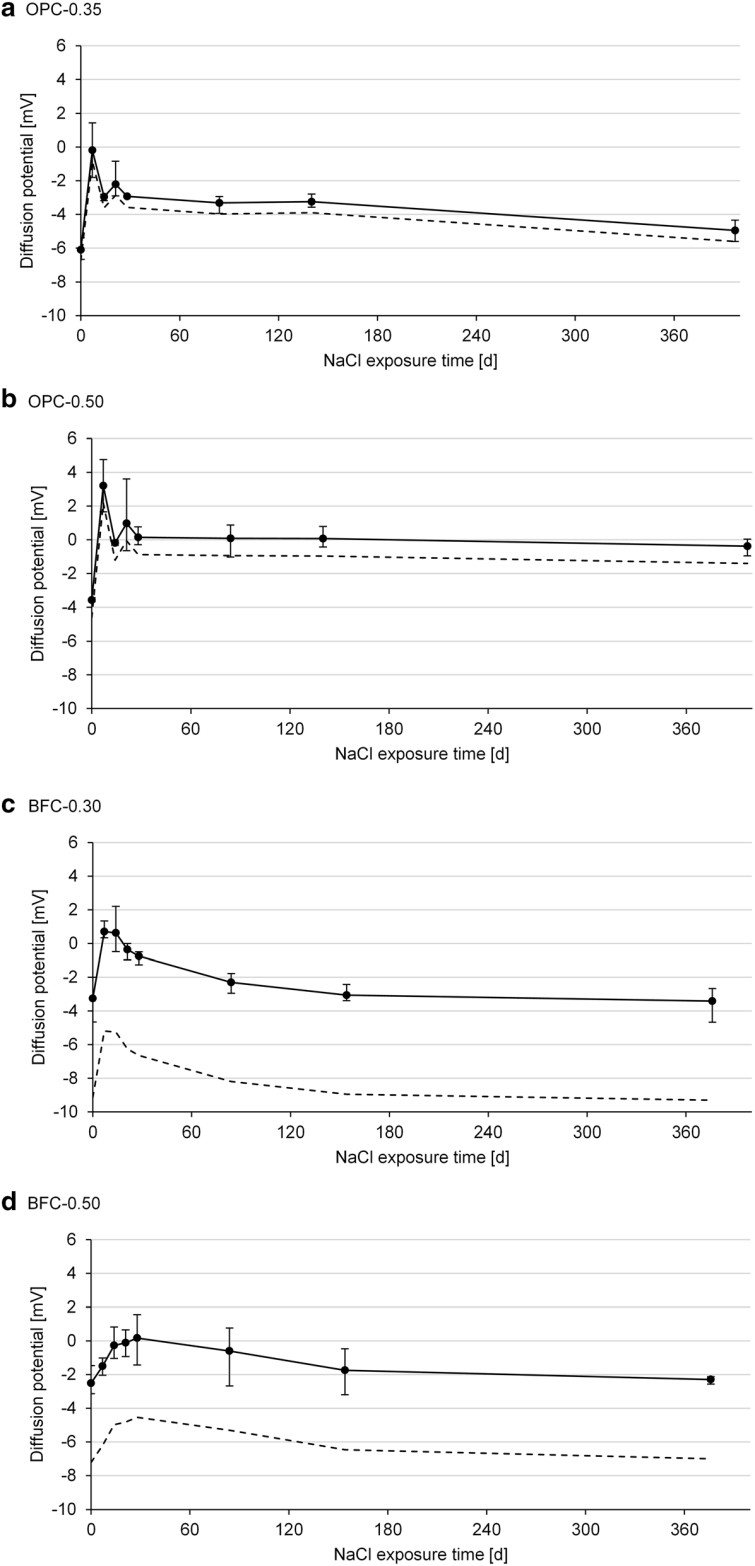
Measured diffusion potentials in the **a**, **b** OPC pastes and **c**, **d** BFC pastes over the NaCl exposure time using the diffusion cell setup; mean, minimum and maximum value of three independent measurements; measured values (full line) and corrected values (dashed line); RE | synthetic pore solution + 1 M NaCl | cement paste | synthetic pore solution | RE

Figure 11 plots the measured diffusion potentials in the cement pastes over the NaCl exposure time. The diffusion potentials range from − 2.5 to − 6.1 mV at the beginning of the NaCl exposure. With progressing chloride penetration, the diffusion potentials increase briefly and then decrease to values of 0 to − 5 mV after 360 d of NaCl exposure.

**Fig. 12:**
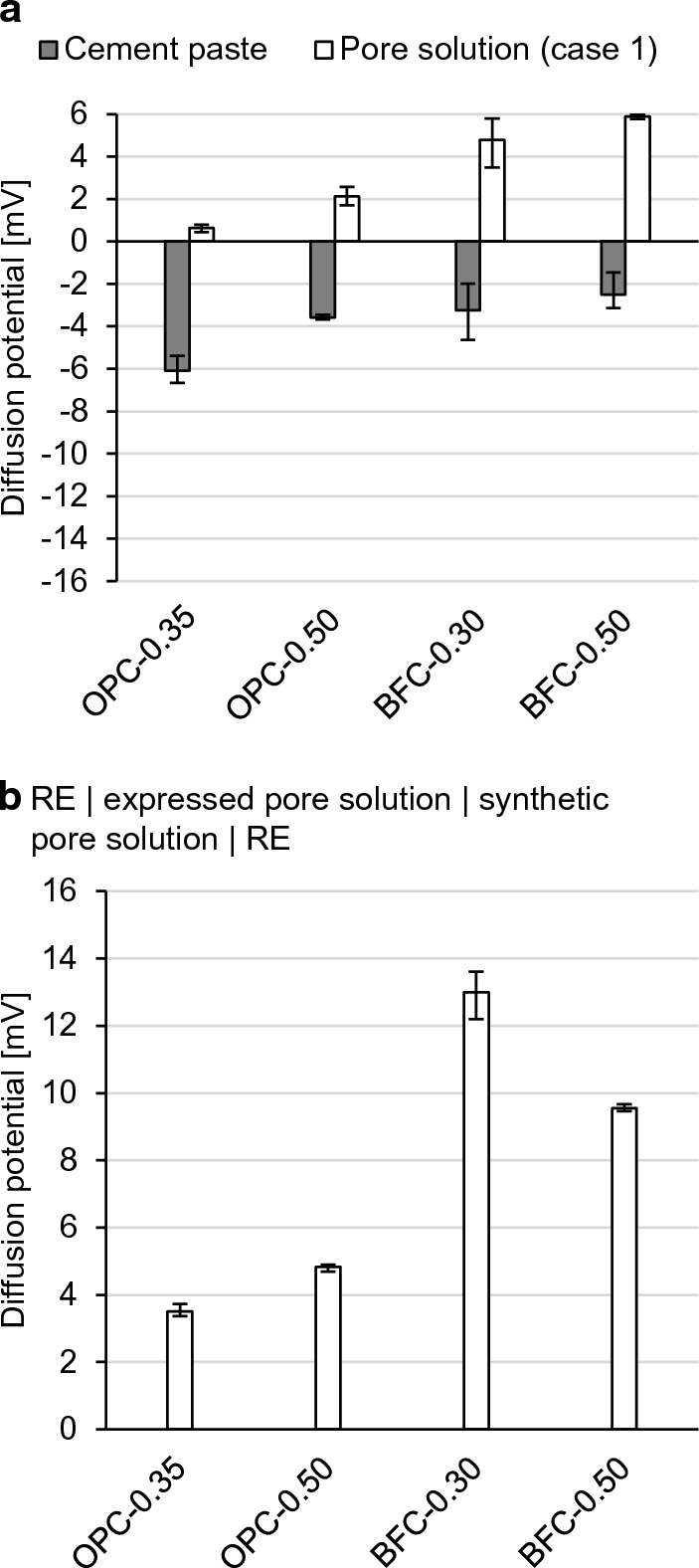
Measured diffusion potentials **a** in pore solution (case 1) and in cement paste, **b** at the junction of the expressed pore solution and the synthetic pore solution using setup 1a; mean, minimum and maximum value of three independent measurements

To determine the contribution of the permselective behavior, the diffusion potentials in the cement pastes are compared with the resulting values in the solution experiments (see Fig. 12). Figure 12a compiles the diffusion potentials from Fig. 11 at the beginning of the NaCl exposure and from Fig. 7a. While negative diffusion potentials in the cement pastes are measured, the values are positive in the pore solution experiments when there are no pH differences (case 1). This shows that the small pH differences of up to 0.3–0.6 units between the expressed and synthetic pore solutions interfere with the measured diffusion potentials.

Figure 12b reproduces the situation when the expressed pore solutions come into contact with the synthetic pore solutions, as for the junction of the cement paste and solution 2 in the diffusion cell setup. Diffusion potentials occur owing to the slight differences in the pore solution compositions. These diffusion potentials measure 3–5 mV for the OPC pore solutions and up to 13 mV for the BFC pore solutions. This corresponds with the higher pH differences of 0.5–0.6 for the BFC pore solutions compared to approx. 0.3 for the OPC pore solutions. It shows that it is challenging to manufacture synthetic pore solutions of identical composition as the corresponding pore solution in the cement paste, particularly for the BFC pore solutions. The BFC pore solutions contain considerable amounts of species, such as sulfide, which are not added to the synthetic pore solutions (see Table 2b). Furthermore, the pore solution compositions (pH, etc.) can change with progressing hydration of the cement pastes (compare [67]). Although the use of synthetic pore solutions minimizes the pH differences, their interference with the diffusion potential cannot be fully eliminated.

For the diffusion potentials studied in the literature [26, 32–35], the pH differences could significantly affect the measurement results when the diffusion cell was used, as noted in [45]. For instance, Elakneswaran et al. [35] measured diffusion potentials of 15–25 mV for OPC pastes with water-cement ratios of 0.40–0.60 using the diffusion cell setup, in which the two compartments were filled with 0.1 and 0.01 M NaCl solution, respectively. With these NaCl solutions, diffusion potentials of approximately 20 mV were included at the junctions of the cement paste and the NaCl solutions, which were in the range of the measured diffusion potentials in the OPC pastes. To measure the diffusion potentials in cement pastes more accurately, it is crucial to minimize the pH differences by using synthetic pore solutions, which closely match the pore solutions in the cement pastes. Even if the chloride gradient was imposed by mixing the chlorides into the cement-based material instead of using the diffusion cell, small pH differences of up to 0.15 units were included [24]. Therefore, the interfering pH differences need to be considered for accurate diffusion potential measurements.

Calculations with Eq. 1 enable the consideration of the interfering pH differences (see Fig. 11 (dashed lines)). The resulting diffusion potentials in the cement pastes are of low magnitude due to the high pH levels of 13–14 in the pore solutions. This shows that the permselective behavior of the cement pastes does not considerably contribute to the diffusion potential as long as the high pH level provides ionic conductivity. Therefore, a significant influence of the permselective behavior on the arising diffusion potential is expected when the pH level is decreased due to carbonation or leaching.

## Conclusions

Diffusion potentials can cause significant errors in corrosion-related investigations of reinforced concrete. Therefore, an improved understanding of the diffusion potentials in cement-based materials is needed. From this experimental study, the following conclusions can be drawn:

- LA-ICP-MS enabled the determination of the profiles of total concentrations of Cl, Na, K and Ca in the cement pastes with high spatial resolution (100 µm). The corresponding profiles of dissolved species concentrations were determined using binding isotherms. This allowed to investigate the permselective behavior of the cement pastes.
- Considerable differences in ion mobilities between Cl^−^ and Na^+^ were found for the BFC pastes, which indicates the permselective behavior of BFC pastes. These differences in ion mobilities were not as pronounced for the OPC paste with a w/c ratio of 0.5, i.e., the permselective behavior is less present. Additional investigations are needed for OPC pastes with a w/c ratio below 0.50 to confirm its permselective behavior.
- The measured diffusion potentials in the presence of chloride profiles were low (− 6 to + 3 mV) for all investigated cement pastes, which was ascribed to the high pH levels (13–14) in the pore solutions. The permselective behavior of the BFC pastes does not considerably contribute to the diffusion potentials as long as these high pH levels provide ionic conductivity.
- When the diffusion cell setup is used, there is a high sensitivity of the measured diffusion potentials in the cement pastes with respect to pH differences. The use of synthetic pore solutions in the diffusion cell compartments minimizes the pH differences at the junction of the cement paste pore solution and the synthetic pore solution. However, these pH differences cannot be completely eliminated. Therefore, the interfering pH differences need to be considered for an accurate measurement of the diffusion potentials in the cement pastes. This can be carried out with the Henderson equation, for which the exact pore solution compositions have to be known.
- For the pore solution experiments, the setups have proven to be suitable for the diffusion potential measurement. The used frit and filter paper did not have permselective behavior, which interferes with the measured diffusion potentials. When the filter paper is used, additional considerations are needed due to some limitations associated with its use (possible contamination of the studied solutions).

Finally, it may be mentioned that in reinforced concrete, often overlapping pH and chloride gradients are present. The pH gradients may result from the leaching of concrete in water or atmospheric carbonation. For these situations, more research is needed to consider the arising diffusion potentials.

## Data Availability

The data that support the findings of this study are available from the corresponding author on request.
